# Regression-based Chinese norms of number connection test A and digit symbol test for diagnosing minimal hepatic encephalopathy

**DOI:** 10.1038/s41598-024-54696-4

**Published:** 2024-02-18

**Authors:** Peng Zhang, Danan Gan, Xiaoling Chi, Dewen Mao, Yueqiu Gao, Yong Li, Daqiao Zhou, Qin Li, Mingxiang Zhang, Bingjiu Lu, Fengyi Li, Jingdong Xue, Xianbo Wang, Hongbo Du, Xiaoke Li, Yijun Liang, Yongan Ye

**Affiliations:** 1 Institute of Liver Disease, Beijing University of Chinese Medicine, Beijing, China; 2 Department of Gastroenterology and Hepatology, Beijing University of Chinese Medicine Affiliated Dongfang Hospital, Beijing, China; 3 Department of Gastroenterology and Hepatology, Institute of Liver Disease, Beijing University of Chinese Medicine Affiliated Dongzhimen Hospital, Beijing, China; 4 Department of Hepatology, Guangdong Provincial Hospital of Chinese Medicine, Guangzhou University of Chinese Medicine, Guangzhou, Guangdong Province China; 5 Department of Hepatology, The First Affiliated Hospital of Guangxi University of Chinese Medicine, Nanning, Guangxi Province China; 6 Department of Hepatology, Shuguang Hospital Affiliated to Shanghai University of Traditional Chinese Medicine, Shanghai, China; 7 Department of Hepatology, The Affiliated Hospital of Shandong University of Traditional Chinese Medicine, Jinan, Shandong Province China; 8 Department of Hepatology, Shenzhen Hospital of Traditional Chinese Medicine, Shenzhen, Guangdong Province China; 9 The Fourth Ward, Fuzhou Infectious Disease Hospital, Fuzhou, Fujian Province China; 10 Department of Integrated Traditional and Western Medicine on Liver Diseases, Shenyang Infectious Disease Hospital, Shenyang, Liaoning Province China; 11 Department of Hepatology, The Affiliated Hospital of Liaoning University of Traditional Chinese Medicine, Shenyang, Liaoning Province China; 12 Treatment and Research Center of Infectious Disease, The Fifth Medical Center of the General Hospital of the People’s Liberation Army, Beijing, China; 13 Department of Hepatology, Shanxi Hospital of Traditional Chinese Medicine, Xi’an, Shanxi Province China; 14 Department of Hepatology, Beijing Ditan Hospital, Beijing, China

**Keywords:** Liver cirrhosis, Minimal hepatic encephalopathy, Neuropsychological test, Standardization, A cross-sectional study, Liver diseases, Encephalopathy, Liver cirrhosis

## Abstract

Number connection test A (NCT-A) and digit symbol test (DST), the preferential neuropsychological tests to detect minimal hepatic encephalopathy (MHE) in China, haven’t been standardized in Chinese population. We aimed to establish the norms based on a multi-center cross-sectional study and to detect MHE in cirrhotic patients. NCT-A and DST were administered to 648 healthy controls and 1665 cirrhotic patients. The regression-based procedure was applied to develop demographically adjusted norms for NCT-A and DST based on healthy controls. Age, gender, education, and age by education interaction were all predictors of DST, while age, gender, and education by gender interaction were predictors of log_10_ NCT-A. The predictive equations for expected scores of NCT-A and DST were established, and Z-scores were calculated. The norm for NCT-A was set as Z ≤ 1.64, while the norm for DST was set as Z ≥ − 1.64. Cirrhotic patients with concurrent abnormal NCT-A and DST results were diagnosed with MHE. The prevalence of MHE was 8.89% in cirrhotic patients, and only worse Child–Pugh classification (*P* = 0.002, OR = 2.389) was demonstrated to be the risk factor for MHE. The regression-based normative data of NCT-A and DST have been developed to detect MHE in China. A significant proportion of Chinese cirrhotic patients suffered from MHE, especially those with worse Child–Pugh classification.

## Introduction

Hepatic encephalopathy (HE) is one of serious complications of liver cirrhosis, severely affecting the lives of patients. Patients with HE have a spectrum of neurologic and psychiatric manifestations, which can be classified into 1–5 categories by the West Haven Criteria^1^. In recent decades, minimal hepatic encephalopathy (MHE), which is the mildest phase of HE, has been added into the West Haven Criteria, attracting more and more attentions^2^. MHE is defined as alterations in psychomotor or neuropsychiatric function without clinical evidence of neurological abnormalities, which can only be identified by neuropsychological or neurophysiological alterations^3,4^. The occurrence of MHE is common in patients with liver cirrhosis and it may affect the quality of life and patients’ driving ability^5–7^. Previous studies also showed that patients with MHE were more likely to develop overt hepatic encephalopathy (OHE), which caused a significant burden to health systems and society overall^8–10^.

Currently, there is no gold standard for the diagnosis of MHE. In western countries, the recognition of MHE depends on Psychometric Hepatic Encephalopathy Score (PHES), Stroop EncephalApp, Animal Naming Test, Critical Flicker Frequency test, and Inhibitory Control Test, et al.^3,4,11,12^. In China, due to the different language and cultural characteristics, abnormal results of both digit symbol test (DST) and number connection test A (NCT-A) have been recommended for diagnosing MHE^13–15^. DST and NCT-A are two sub-tests of PHES, and the normative data based on Chinese population are still lacking^3,4,11^. While China is a country with a vast territory and a large population, previous attempts on norms for NCT-A and DST were mostly based on studies with a small simple size and a single center, lacking consideration for demographic variables, such as age, gender, and education level, et al.^16–19^. Therefore, it is necessary to develop new norms for NCT-A and DST in China, which should be adjusted by demographic variables and standardized based on a multi-center, large sample size study.

The present cross-sectional study aimed to establish the Chinses norms for NCT-A and DST based on a large sample size of healthy population from multiple centers. The prevalence of MHE in patients with liver cirrhosis was further investigated in accordance with the established norms.

## Methods

### Healthy controls

648 Chinese-speaking healthy individuals aged 18–75 from 12 centers of China (Beijing University of Chinese Medicine Affiliated Dongzhimen Hospital (Beijing), Beijing Ditan Hospital (Beijing), The Fifth Medical Center of the General Hospital of the People’s Liberation Army of China (Beijing), Fuzhou Infectious Disease Hospital (Fuzhou, Fujian), Guangdong Provincial Hospital of Chinese Medicine (Guangzhou, Guangdong), Shenzhen Hospital of Traditional Chinese Medicine (Shenzhen, Guangdong), the First Affiliated Hospital of Guangxi University of Chinese Medicine (Nanning, Guangxi), the First Affiliated Hospital of Liaoning University of Traditional Chinese Medicine (Shenyang, Liaoning), the Affiliated Hospital of Shandong University of Traditional Chinese Medicine (Jinan, Shandong), Shanxi Provincial Hospital of Traditional Chinese Medicine (Xi’an, Shanxi), Shenyang Infectious Disease Hospital (Shenyang, Liaoning), Shuguang Hospital Affiliated to Shanghai University of Traditional Chinese Medicine (Shanghai)) were consecutively recruited from December 2016 to February 2018. Participation was voluntary, and participants from both urban and rural areas were recruited with a basic knowledge of numbers. Eligible healthy controls were screened according to the following exclusion criteria: (1) subjects with chronic liver disease, neurological or psychiatric disease, or other diseases that can impair cognitive function; (2) use of psychoactive drugs; (3) alcohol consumption greater than 40 g/day for males and 20 g/day for females; (4) pregnant and lactating women; and (5) inability to read and write. Following their informed consent, all individuals underwent the NCT-A and DST tests, and their demographic data were collected. Those with abnormal results of both NCT-A and DST and with critical missing data were also excluded during statistical analysis.

### Patients with liver cirrhosis

1665 cirrhotic patients aged 18–75 from the above mentioned 12 medical centers of China were consecutively enrolled in this study between December 2016 and June 2018. Each patient provided written informed consent. Liver cirrhosis was diagnosed according to the clinical guidelines^20,21^. Both outpatients and hospitalized patients of a single specific etiology were included, except cirrhotic patients caused by alcoholic liver disease. After neuropsychological tests, patients’ demographic and clinical data within 2 weeks were collected. The following exclusion criteria were applied for cirrhotic patients: (1) patients with OHE or a past history of OHE; (2) patients with other severe complications, including bleeding from esophageal and gastric varices and severe infection; (3) patients with neurological or psychiatric disease, or other diseases that can impair cognitive function; (4) patients with hepatocellular carcinoma or other malignancy; (5) use of lactulose, antibiotics, or psychoactive drugs; (6) pregnant and lactating women; and (7) inability to read and write. Patients with critical missing data were excluded during statistical analysis.

The study was conducted following the guidelines of the Declaration of Helsinki (as revised in 2013) and was approved by the institutional review board of each center (namely Beijing University of Chinese Medicine Affiliated Dongzhimen Hospital (Beijing), Beijing Ditan Hospital (Beijing), The Fifth Medical Center of the General Hospital of the People’s Liberation Army of China (Beijing), Fuzhou Infectious Disease Hospital (Fuzhou, Fujian), Guangdong Provincial Hospital of Chinese Medicine (Guangzhou, Guangdong), Shenzhen Hospital of Traditional Chinese Medicine (Shenzhen, Guangdong), the First Affiliated Hospital of Guangxi University of Chinese Medicine (Nanning, Guangxi), the First Affiliated Hospital of Liaoning University of Traditional Chinese Medicine (Shenyang, Liaoning), the Affiliated Hospital of Shandong University of Traditional Chinese Medicine (Jinan, Shandong), Shanxi Provincial Hospital of Traditional Chinese Medicine (Xi’an, Shanxi), Shenyang Infectious Disease Hospital (Shenyang, Liaoning), and Shuguang Hospital Affiliated to Shanghai University of Traditional Chinese Medicine (Shanghai)).

### Neuropsychological tests

Healthy controls and cirrhotic patients were required to complete NCT-A test by drawing lines sequentially connecting numbers, from 1 to 25, as quickly and accurately as possible. Numbers are randomly distributed on a printed A4-size paper. If an error was made, the participant would be asked to restart from where the error happened. The time of completion was calculated as the score for NCT-A.

When completing the DST test, healthy controls and cirrhotic patients were provided a sheet with nine unique symbols, each paired with numbers ranging from 1 to 9. The remainder of the paper consists of randomized numbers and blank squares below. Participants were required to draw the corresponding symbols in the blank squares as quickly as possible. The test is timed, and the score for DST is defined as the total number symbols of correctly paired items in 90 s.

NCT-A and DST were conducted by two trained examiners. Before the tests, there was a practice to help the participant understand the process. Patients with abnormal results of both NCT-A and DST were diagnosed as MHE.

### Statistical analysis

Normally distributed data were expressed as means ± standard deviation (SD), while data obeying abnormal distribution were presented as median (interquartile range). The χ^2^ or Fisher’s exact test was used for categorical variables, and the Mann–Whitney U test or analysis of variance was conducted assess the difference of continuous variables.

The method to develop the regression-based normative data of NCT-A and DST referred to the previously described procedure^22–24^. Spearman correlations between the scores of DST and NCT-A and the demographic variables (age, gender, and education) were computed. The multivariate linear regression model was conducted to find predictors of neuropsychological tests. Age, age^2^, education, education^2^, gender, and all two-way interactions between these variables, were included as potential predictors. Age and education were centered (age = calendar age in years—mean age in healthy controls; education = years of education—mean years of education in healthy controls) before computing quadratic terms and interactions to avoid multicollinearity. Squared terms of age and years of education were added in the model to allow for quadratic effects between these independent variables and the scores of neuropsychological tests. Gender was coded as male = 1 and female = 2. The following assumptions were tested: (1) homoscedasticity was evaluated via plots of regression predicted values to residuals values; (2) normality of the standardized residuals was evaluated by using Q-Q plots and histograms of residual values; (3) The occurrence of multicollinearity was evaluated by computing the variance inflation factor (VIF), which should not be greater than 10; and (4) potential influential cases were evaluated by computing Cook’s distances. The final multivariate linear regression model was obtained using the stepwise procedure.

The regression-based normative data adjusted for demographic variables was established by means of a four-step procedure: (1) the expected scores of NCT-A and DST were computed based on the established multivariate linear regression model: Y = B_0_ + B_1_*X_1_ + B_2_*X_2_ + ⋯ + B_n_*X_n_, with B_0_ = the intercept, B_n_ = the regression weight(s), and X_n_ = the predictor values; (2) the residual value was computed based on a subtraction between the raw score and the predicted score (*e*_i_ = raw scores – predicted scores); (3) standard deviation (SD*e*) of the residuals was acquired in the regression model, and the residual is standardized: Z_i_ = *e*_i_/SD_*e*_; and (4) the standardized residuals (Z-scores) were transformed into percentiles referring to the standard normal distribution of the residuals.

To find the relevant factors for the prevalence of MHE, total bilirubin, albumin, platelets, prothrombin activity, ammonia, ascites at study inclusion, and Child–Pugh classification were included in the univariate logistic analysis or the χ^2^ or Fisher’s exact test. Variables with *P* values < 0.10 were entered into a multivariable logistic regression model after adjusting for age, gender, and education. The Cochran–Armitage trend test was further conducted to analyze the prevalence of MHE among patients with Child–Pugh class A, B, and C. All statistical analyses were two-tailed at the 5% level. All analyzes were performed using SPSS version 23 (IBM Corp., Armonk, NY).

## Results

### Characteristics of healthy controls and cirrhotic patients

In total, 648 healthy controls and 1665 cirrhotic patients were included. The mean age of healthy controls was 35 (28, 50) years and 43.67% were male, while the average age of cirrhotic patients was 53 (45, 61) years and males made up 70.33%. The length of education in healthy controls was 16 (12, 16) years, and it was 9 (9, 12) years in cirrhotic patients. Healthy controls’ performance of both NCT-A and DST were significantly better than cirrhotic patients’. The detailed demographic characteristics and neuropsychological raw scores were reported in Table 1.

**Table 1:** Demographic, clinical and neuropsychological raw scores of healthy controls and cirrhotic patients.

Variable	Healthy controls (*n* = 648)	Cirrhotic patients (*n* = 1665)	*P* value
Age (years)	35 (28, 50)	53 (45, 61)	0.000
Male gender, n (%)	283 (43.67%)	1171 (70.33%)	0.000
Education (years)	16 (12, 16)	9 (9, 12)	0.000
BMI, (kg/m^2^)	22.21 (20.08, 24.60)	23.25 (21.30, 25.35)	0.000
NCT-A raw score (second)	44 (37, 56)	60 (50, 79)	0.000
DST raw score (point)	56.92 ± 14.18	41 (32, 50)	0.000
Aetiology of cirrhosis			
Hepatitis B, n (%)	–	1446 (86.85)	–
Hepatitis C, n (%)	–	91 (5.47%)	–
Autoimmune, n (%)	–	109 (6.55)	–
Drug-induced liver injury, n (%)	–	18 (1.08)	–
Child–Pugh A/B/C, n (%)	–	1088 / 365 / 102 (69.97 / 23.47 / 6.56)	–
Duration of cirrhosis (years)	–	2 (1, 5)	–

BMI, body mass index; DST, digit symbol test; IQR, interquartile range; SD, standard deviation; NCT-A, number connection test-A. Normally distributed data were expressed as means ± SD, while data obeying abnormal distribution were presented as median (IQR).

### Effects of demographic factors on NCT-A and DST in healthy controls

**Figure 1:**
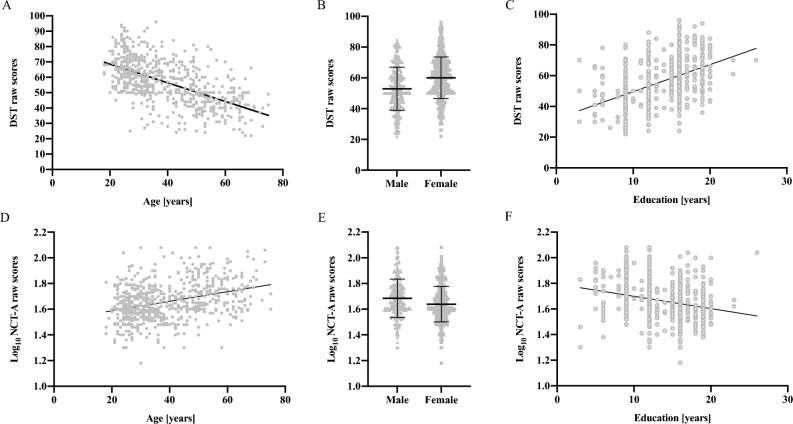
Distribution of NCT-A and DST raw scores in healthy controls, according to age (**A** and **D**), gender (**B** and **E**), and education (**C** and **F**). The dots represent individual values and the bars indicate the mean and standard deviation. NCT-A, number connection test-A; DST, digit symbol test.

The raw data of DST were normally distributed, and the scores of NCT-A were log transformed to fit the Gaussian distribution, or, at least, to reduce their positive skew. Spearman correlations showed that, as age increases, the scores of DST decrease (r = − 0.591, *P* = 0.000) and log_10_ NCT-A increase (r = 0.326,* P* = 0.000). By contrast, as the education years increase, the scores of DST increase (r = 0.472, *P* = 0.000) and log_10_ NCT-A decrease (r = − 0.263, *P* = 0.000). Females had better performance in both NCT-A and DST. The distributions of log_10_ NCT-A and DST scores in different age, gender, and education were shown in Fig. 1.

The assumptions of multivariate linear regression analysis were met in both models. The VIF values were all below the threshold of 10, which meant that there was no multicollinearity (Table 2). The maximum Cook’s distance values were 0.057 and 0.31, respectively, suggesting no influential cases. The visual analysis of the Q–Q normality diagrams and histograms of the residual values for the final models fulfilled the assumption of normality, and plots of regression predicted values to residuals values ensured the assumption of homoscedasticity was not violated. (Supplementary Figure).

**Table 2:** Multiple linear regression models for the NCT-A and DST in healthy controls.

Test	Predictor	*B*	SE	*β*	*t*	*P*	VIF	Adjusted *r*^2^
NCT− A	Intercept	1.701	0.018		95.302	0.000		0.130
	Age (y)	0.003	0.000	0.270	6.248	0.000	1.389	
	Gender*	− 0.026	0.011	− 0.090	− 2.406	0.016	1.043	
	Interaction (Education (y) * Gender)	− 0.003	0.001	− 0.106	− 2.475	0.014	1.356	
DST	Intercept	50.357	1.477		34.105	.000		0.393
	Age (y)	− 0.477	0.039	− 0.450	− 12.319	0.000	1.421	
	Gender*	3.682	0.895	0.129	4.114	0.001	1.045	
	Education (y)	0.973	0.138	0.258	7.075	0.000	1.417	
	Interaction (Age (y) * Education (y))	− 0.032	0.01	− 0.107	− 3.237	0.001	1.169	

*Male is calculated as 1, and female is calculated as 2.

DST, digit symbol test; SE, standard error; NCT-A, number connection test-A; VIF, variance inflation factor.

The multivariate linear regression model was conducted. Age, gender, education, and age by education interaction were all predictors of DST, while age, gender, and education by gender interaction were predictors of log_10_ NCT-A (Table 2).

### The normative data of NCT-A and DST in healthy controls

The predictive equations for expected scores of NCT-A and DST were calculated as follows: predicted DST = 50.357−0.477 * (calendar age—39.19) + 0.973 * (years of education − 14.16) + 3.682 * gender -0.032 * (calendar age—39.19) * (years of education − 14.16), and predicted log_10_ NCT-A = 1.701 + 0.003 * (calendar age—39.19) − 0.026 * gender − 0.003 * gender * (years of education − 14.16). SD*e* was 11.02 for DST, and 0.13 for log_10_ NCT-A, respectively. Z-scores for NCT-A and DST = (measured value−expected value)/standard deviation of the residuals. Finally, according to the conversion tables of Z-score^25^, the norm for NCT-A was set as Z ≤ 1.64, while the norm for DST was set as Z ≥ − 1.64.

### A user-friendly normative data calculator

A user-friendly, interactive normative data calculator in an Excel work-sheet was developed (Supplementary file 1) to compute Z-scores of DST and NCT-A for Chinese population aged 18–75. When we input the raw score of NCT-A or DST, age, gender, and education years, the calculator will automatically show the corresponding Z-score.

For example, for a 70 years old male with 16 years of education whose raw score of DST is 30, the Z-score for DST will be calculated and shown as − 0.85.

### Prevalence and characteristics of MHE

**Figure 2:**
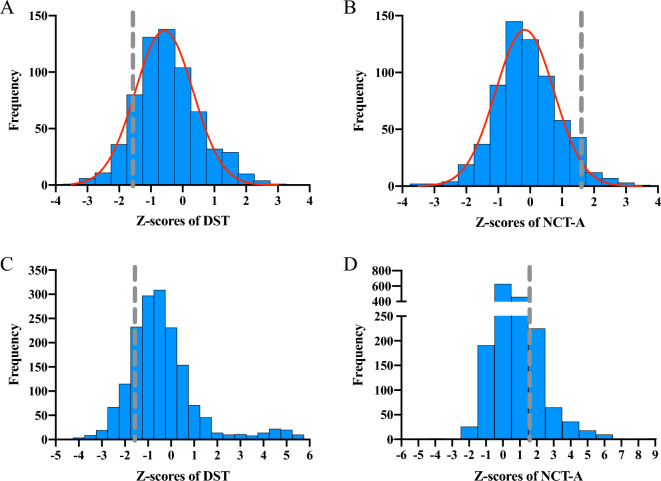
Frequency distribution on Z-scores of NCT-A and DST in healthy controls (**A** and **B**) and cirrhotic patients (**C** and **D**). A. Z-scores of NCT-A in healthy controls. B. Z-scores of DST in healthy controls. C. Z-scores of NCT-A in cirrhotic patients. D. Z-scores of DST in cirrhotic patients. The gray dashed line in each figure depicts the norms for Z-scores of NCT-A and DST, respectively. NCT-A, number connection test-A; DST, digit symbol test.

Compared with healthy controls, cirrhotic patients had significantly higher Z-scores for log_10_ NCT-A and lower Z-scores for DST. (Fig. 2) Criteria of NCT-A and DST for MHE were set as Z ≤ 1.64 (NCT-A) and Z ≥ − 1.64 (DST), after entering patients’ age, gender, and education, into Z-scores computing formula. In total, 148 cirrhotic patients with concurrent abnormal NCT-A and DST results were diagnosed with MHE. The prevalence of MHE was 8.89% in patients with liver cirrhosis, which differed among patients with Child–Pugh classification (CPC) A (79/1088, 7.26%), B (38/365, 10.41%), and C (17/102, 16.67%). Compared with patients without MHE, patients with MHE were significantly older, and had a higher proportion of female, a lower level of education, and a higher proportion of HCV infection, autoimmune liver diseases, and drug-induced liver injury. Patients with MHE also had a lower ALB level and a higher proportion of ascites (Table 3). By using the univariate logistic analysis and the chi square test, CPC and other related variables, such as venous ammonia, were included in the multivariable logistic regression model to find correlated factors with the prevalence of MHE. Finally, only worse CPC was demonstrated to be the risk factor for MHE (*P* = 0.006, OR = 2.389), and the Cochran–Armitage trend test confirmed a linear trend between CPC grading and the prevalence of MHE (*P* = 0.001). (Table 4) There was no correlation between MHE and laboratory examinations.

**Table 3:** Demographic and biochemical data of cirrhotic patients with and without MHE.

Variable	Patients with MHE (n = 148)	Patients without MHE (n = 1517)	*P* value
Age (years)	56 (47.25, 62.75)	52 (45, 61)	0.008
Male gender, n (%)	72 (48.65)	1099 (72.45)	0.000
Education (years)	9 (6, 12)	9 (9, 12)	0.000
Aetiology of cirrhosis			0.025
Hepatitis B, n (%)	117 (79.05)	1329 (87.61)	
Hepatitis C, n (%)	12 (8.11)	79 (5.21)	
Autoimmune, n (%)	14 (9.46)	95 (6.26)	
Drug-induced liver injury, n (%)	4 (2.70)	14 (0.92)	
Child–Pugh A/B/C, n (%)	79 / 38 / 17 (58.96 / 28.36 / 12.69)	1009 / 327 / 85 (71.01 / 23.01 / 5.98)	0.002
Duration of cirrhosis (years)	2 (1, 4)	2 (1, 5)	0.325
NCT-A raw score (second)	121 (101.25, 171.25)	59 (49, 72)	0.000
DST raw score (point)	20.5 (16, 24.75)	42 (34, 50)	0.000
Total bilirubin (umol/L)	20.90 (12.80, 35.60)	20.10 (13.80, 33.00)	0.686
Albumin (g/L)	37.02 ± 7.32	39.50 (33.20, 44.80)	0.013
Platelets (*10^9^/L)	103.00 (63.75, 162.25)	113.00 (69.00, 168.00)	0.360
Prothrombin activity (%)	80(63.5, 92)	82 (65, 95)	0.254
Ammonia (umol/L)	31.65 (27.50, 49.75)	35.00 (22.00, 52.75)	0.716
Ascites at study inclusion, n (%)	39 (36.11)	283 (25.84)	0.022

DST, digit symbol test; MHE, minimal hepatic encephalopathy; NCT-A, number connection test-A.

**Table 4:** Univariate and multivariate logistic regression models for MHE.

Variable	Univariate logistic regression model			Multivariate logistic regression model		
	Odds ratio	95% Wald confidence limits	*P* value	Odds ratio	95% Wald confidence limits	*P* value
Age (years)	1.019	1.003–1.035	0.021	0.996	0.965–1.028	0.813
Female gender, n (%)	2.102	2.960–1.492	0.000	–	–	–
Education (years)	0.903	0.865–0.943	0.000	0.928	0.849–1.016	0.106
Aetiology of cirrhosis	–	–	0.074	–	–	0.647
Hepatitis B, n (%)	–	–	–	–	–	–
Hepatitis C, n (%)	–	–	–	–	–	–
Autoimmune, n (%)	–	–	–	–	–	–
Drug-induced liver injury, n (%)	–	–	–	–	–	–
Child–Pugh A/B/C, n (%)	1.564	1.209–2.025	0.001	2.389	1.279–4.463	0.006
Duration of cirrhosis (years)	0.994	0.955–1.033	0.747	–	–	–
Total bilirubin (umol/L)	1.000	0.997–1.003	0.988	–	–	–
Albumin (g/L)	0.974	0.953–0.995	0.018	1.025	0.962–1.092	0.446
Platelets (*10^9^/L)	1.000	0.998–1.002	0.925	–	–	–
Prothrombin activity (%)	0.998	0.994–1.003	0.536	–	–	–
Ammonia (umol/L)	1.004	0.990–1.018	0.577	1.000	0.985–1.016	0.974

## Discussion

Chinese guidelines have recommended NCT-A and DST as preferential neuropsychological tests to detect MHE in Chinese cirrhotic patients^15,26^. Possible deficits in attention, memory, processing speed, and executive function can be detected, which are common and prominent in MHE. Despite the importance for the clinical screening of MHE, they have not been standardized in China yet. As far as we know, this is the first time to develop the regression-based normative data of NCT-A and DST in China based on a multi-center study. Based on the normative data, 8.89% of cirrhotic patients were diagnosed with MHE and worse CPC was found to be the risk factor.

Among the methods for developing the normative data, the use of mean and standard deviation within each subgroup, and conversion of raw scores to metrics such as Z or T scores were the most commonly used, considering the influence of demographic variables (e.g. age, education, gender) on the performance of neuropsychological tests. The use of mean and standard deviation requires dividing the sample into subgroups according to the predictive variables, and as a result, the sample size within subgroups will be significantly reduced. We have tried to establish the norms for NCT-A and DST using the mean and standard deviation, and the sample of some subgroups stratified by age, gender, and education was too small to ensure the reliability of the norms^27^. Therefore, the present study developed the regression-based normative data by converting raw scores to Z-scores. Some other studies have tried to establish the norms for NCT-A and DST in China. However, they just included a small sample of healthy participants in one or two medical centers, and ignored the influence of some demographic variables^16–19^. Healthy controls from 12 medical centers in different parts of China were included in the present study, ensuring the representativeness of participants to Chinese population. The results of the multivariate linear regression model showed that age, gender, education, and age by education interaction were predictors of DST, while age, gender, and education by gender interaction were predictors of log_10_ NCT-A. Younger age and more education years were reported to be associated with better performance of NCT-A and DST in most previous normative studies^28,29^. The processing speed of healthy individuals tends to decline along with the growth of age^30^. Moreover, participants with more educational experience are more familiar with paper–pencil tests than those without. The association between the scores of NCT-A and DST, and gender was a controversial issue in previous studies. While no significant difference was reported in some studies^31–34^, females were shown to possess better visual processing speed than males in the others^19,29,35–37^. Our results also showed that females outperformed males in both NCT-A and DST. Based on the above results, the normative data of NCT-A and DST adjusted to age, gender, education and interactions were developed, and a user-friendly EXCEL sheet was created for clinicians and patients. Specifically, results of NCT-A and DST in elderly people will be analyzed in future, which will not be described in this paper.

According to the regression-based normative data of NCT-A and DST, the prevalence of MHE in cirrhotic patients was 8.89% in China. It was lower than those in previous studies with similar diagnosis criteria. A positive correlation between the prevalence of MHE and patients’ CPC was shown, which was consistent with previous reports^13,16,38^. The low prevalence may be caused by following reasons. Firstly, our study did not include cirrhotic patients caused by alcoholic liver disease, as well as patients with a history of OHE. There is mounting evidence that alcohol may impair patients cognitive function^39,40^, which can bring cofounding factors to the diagnosis of MHE. Furthermore, the proportion of cirrhotic patients with CPC-B/C in our study was lower than that in previous studies, whom were reported to have a higher prevalence of MHE than patients with CPC-A. The different normative data of NCT-A and DST also accounted for the different prevalence.

Our study was not without limitations. Healthy controls included were not from community, but were mostly patients’ family members. Healthy controls’ average age was younger than the reported average age of Chinese^41^. Moreover, medical centers in our study were mostly located in key cities. This may affect the representativeness of healthy controls. However, patients and their family members in large hospitals usually come not only from local cities, but also from all over the country. The regression-based approach was also conducted to reduce bias. Lastly, although the demographic variables were considered in developing the regression-based normative data, occupation and the urban–rural difference were not taken into account. Patients with some jobs were difficult to be classified as “white collars” or “blue collars” workers, and occupation was also closely related to the education level. With the rapid development of China in recent decades, it has been difficult to distinguish whether people come from urban or rural areas.

## Conclusion

Our study firstly developed the regression-based normative data of NCT-A and DST based on a large sample from 12 medical centers all over the country, and a user-friendly calculator was offer. A considerable number of cirrhotic patients were diagnosed with MHE based on the normative data, and the prevalence was positively correlated with CPC. The standardization of NCT-A and DST will contribute to the clinical and research practice in China. The clinical screening of MHE needs to be paid attention in cirrhotic patients, especially those with CPC-B/C.

### Supplementary Information

Supplementary Information.

Supplementary Figure S1.

Supplementary Information.

## Data Availability

The data used to support the findings of this study are available from the corresponding author upon request.

## Abbreviations

HE: Hepatic encephalopathy
MHE: Minimal hepatic encephalopathy
OHE: Overt hepatic encephalopathy
PHES: Psychometric hepatic encephalopathy score
DST: Digit symbol test
NCT-A: Number connection test A
SD: Standard deviation
CPC: Child–Pugh classification
HCV: Hepatitis C virus
