# Supplementary material for: Regression-based Chinese norms of number connection test A and digit symbol test for diagnosing minimal hepatic encephalopathy
Source: Sci Rep. 2024 Feb 18;14:4005. doi: 10.1038/s41598-024-54696-4 (PMC10874952; doi:10.1038/s41598-024-54696-4)
Supplement: Supplementary file 1 — Supplementary Information. [file 41598_2024_54696_MOESM1_ESM.docx]

**Supplementary Figure.** To explore the effects of demographic factors on NCT-A and DST in healthy controls, the normality of standardized residuals in the multiple linear regression models was evaluated by using Q-Q plots and histograms of residual values (A, B, D, and E). The homoscedasticity was evaluated via plots of regression predicted values to residuals values (C and F). NCT-A, number connection test-A; DST, digit symbol test.
