# Supplementary figures and images for: Regression-based Chinese norms of number connection test A and digit symbol test for diagnosing minimal hepatic encephalopathy
Source: Sci Rep. 2024 Feb 18;14:4005. doi: 10.1038/s41598-024-54696-4 (PMC10874952; doi:10.1038/s41598-024-54696-4)

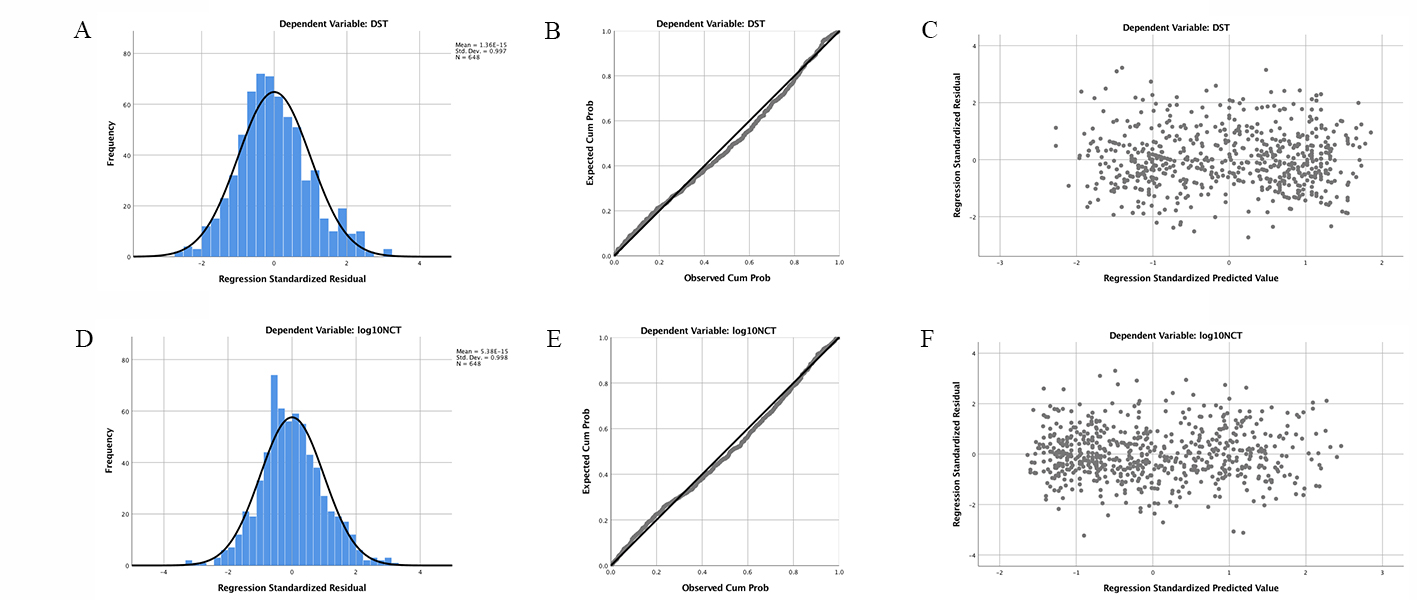

Supplement: Supplementary file 2 — Supplementary Figure S1. [file 41598_2024_54696_MOESM2_ESM.jpg]
